# Cancer and aging: complex associations and therapeutic targets

**DOI:** 10.1186/s43556-026-00423-6

**Published:** 2026-03-31

**Authors:** Jingjing Liang, Siying Zang, Zhe Wang, Ruijuan Zhang

**Affiliations:** 1 The First Clinical Medical College, Shanxi Medical University, Taiyuan, 030001 Shanxi China; 2 Department of Hematology, the First Hospital of Shanxi Medical University, Taiyuan, 030001 Shanxi China; 3 The Third Clinical Medical College, Shanxi Medical University, Taiyuan, 030001 Shanxi China; 4 Department of Hematology, the Third Hospital of Shanxi Medical University, Shanxi Bethune Hospital, Shanxi Academy of Medical Sciences, Tongji Shanxi Hospital, Taiyuan, Shanxi 030002 China; 5 Department of Gynecology, the First Hospital of Shanxi Medical University, Taiyuan, 030001 Shanxi China; 6 Research Center for Hemostatic Disorders and Hematologic Malignancies, Shanxi Medical University, Taiyuan, 030001 Shanxi China

**Keywords:** Aging, Cancer, Hallmark, Molecular linkage, Therapeutic target

## Abstract

The incidence of cancer increases markedly with aging, and the two processes share underlying molecular mechanisms. In the context of global population aging and rising cancer incidence, nine convergent hallmark axes have been identified: genomic instability, epigenetic drift, inflammation–immunity imbalance, microbiome dysbiosis, metabolic reprogramming, telomere attrition, stem cell exhaustion, cellular senescence, and autophagy dysfunction. These hallmarks constitute an integrated regulatory network that operates synergistically, antagonistically, or through bidirectional feedback across molecular, cellular, and microenvironmental levels. Genomic instability, epigenetic remodeling, chronic inflammation, microbiome dysbiosis, and metabolic reprogramming in aging often act synergistically to promote tumorigenesis, whereas telomere attrition and stem cell exhaustion primarily exert antagonistic, tumor-suppressive effects. Cellular senescence and autophagy dysfunction display context-dependent dual roles. Importantly, this network framework has direct relevance to cancer therapeutics. Although chemotherapy, radiotherapy, and immunotherapy effectively suppress tumor progression, they frequently induce therapy-induced senescence, characterized by cell-cycle arrest and a senescence-associated secretory phenotype, thereby accelerating functional decline and increasing long-term toxicities in older patients. The proposed “synergistic–antagonistic–dual” framework linking aging and cancer not only helps explain the disproportionate cancer burden in older adults but also supports a “one drug, two targets” therapeutic paradigm. Targeting these shared pathways has delayed aging phenotypes and suppressed tumorigenesis in preclinical studies and early clinical trials, highlighting the potential of integrated interventions that concurrently address aging and cancer.

## Introduction

Aging is a progressive, time-dependent decline in physiological function, characterized by a gradual loss of structural integrity and functional capacity across molecular, cellular, tissue, and organ levels [1]. In addition to its intrinsic biological processes, aging plays a central role in the onset and progression of numerous chronic noncommunicable diseases [2]. Global population aging is currently accelerating at an unprecedented rate. According to United Nations projections, the number of individuals aged ≥ 65 years is expected to increase from 703 million in 2019 to 1.5 billion by 2050, accounting for approximately 16% of the global population [3, 4]. This demographic transition will substantially increase the burden of age-related diseases and pose major challenges to public health systems worldwide.

Cancer has emerged as a leading contributor to the global disease burden and represents a major component of the age-related disease spectrum. In 2022, nearly 20 million new cancer cases were diagnosed worldwide, accompanied by ≥ 9.7 million cancer-related deaths [5]. Driven by the combined effects of population growth and demographic aging, annual cancer incidence is projected to exceed 35 million by 2050, representing a 77% increase compared with 2022 levels [6]. Epidemiological data indicate that more than half of all cancers are diagnosed in individuals aged ≥ 65 years, with both incidence and mortality rates peaking among those aged ≥ 80 years, i.e., the highest across all age groups [7, 8].

These data demonstrate that older adults experience the highest incidence of malignancy and bear the greatest burden of cancer-related mortality, with neoplastic disease representing a major threat to healthspan. Accordingly, elucidating the intrinsic relationship between aging and malignant transformation is of substantial scientific and clinical importance.

In this review, established “hallmark” frameworks for aging and cancer serve as the analytical foundation. We synthesize current evidence to delineate the integrated molecular, cellular, and microenvironmental networks through which these processes intersect, with particular emphasis on one-to-one correspondences between aging hallmarks and key events driving carcinogenesis. Building on this framework, we critically evaluate anticancer strategies informed by the biology of aging. By integrating recent advances, we aim to provide a coherent theoretical framework and a practical roadmap for precision prevention and effective cancer management in older adults.

## Aging and cancer as intertwined processes

The clinical significance of aging and cancer has driven researchers to explore their underlying mechanisms and characteristics as well as their hallmarks. Aging and cancer are not distinct biological endpoints but interconnected life processes driven by shared intrinsic mechanisms. Although the intrinsic factors governing their crosstalk have been long discussed, many questions remain unresolved and warrant further investigation. Here, we integrated the hallmarks of aging and cancer and discussed their connections to address relevant clinical phenomena.

### Hallmarks of aging and cancer

Systematic research on aging and cancer has led to comprehensive frameworks describing their respective hallmarks [9–11]. Comparative analyses indicate that 10 cancer hallmarks, excluding invasion and metastasis, angiogenesis, evasion of growth-inhibitory signals, and sustained proliferative signaling, closely align with 12 hallmarks of aging at the molecular level. This substantial overlap underscores that aging and cancer are not isolated phenomena but are embedded within interconnected, reciprocally modulatory networks rooted in shared molecular mechanisms.

**Fig. 1:**
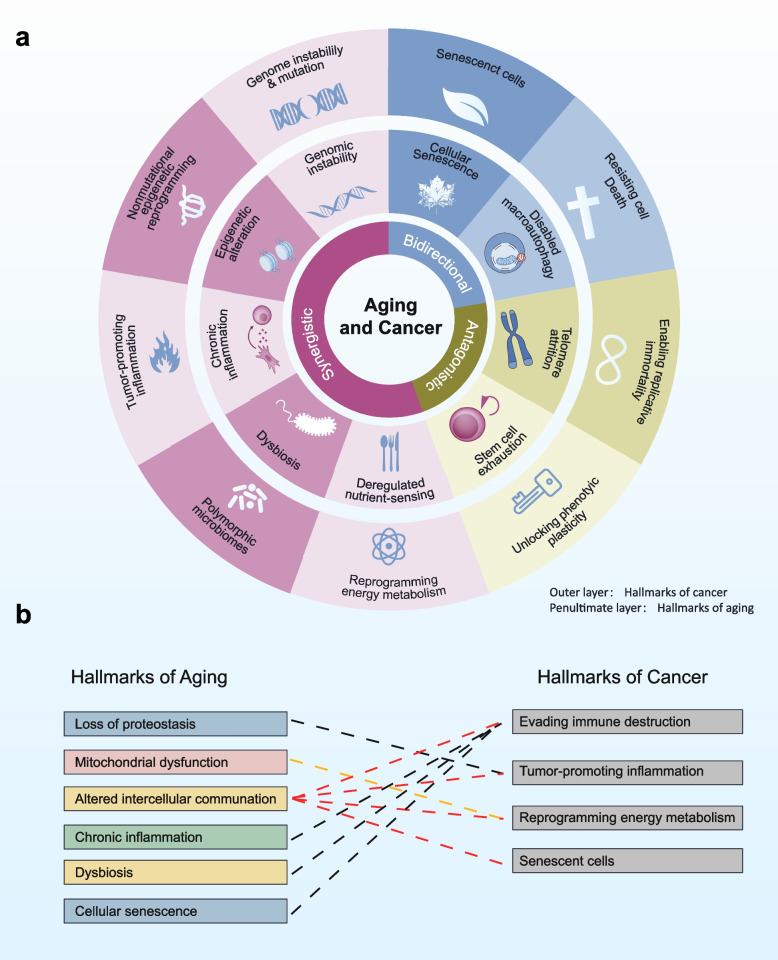
Mapping aging and cancer hallmarks. (a) One-to-one correspondences: nine hallmark pairs show direct relationships between aging and cancer. (b) Non-correspondent connections

Among these hallmarks, nine exhibit direct one-to-one correspondence (Fig. 1a). Their combined effects on cancer evolution can be classified into three functional modalities: synergistic, antagonistic, and bidirectional. Genomic instability, epigenetic remodeling, chronic inflammation, microbiome dysbiosis, and metabolic reprogramming act synergistically to promote oncogenesis. Conversely, telomere attrition and stem cell exhaustion primarily exert antagonistic, tumor-suppressive effects. Cellular senescence and autophagic dysfunction display context-dependent behavior; under certain microenvironmental conditions, they restrain tumor growth, whereas in others they accelerate tumor progression, reflecting a dual biological role.

In addition to these nine paired hallmarks, several non-corresponding aging traits converge to influence carcinogenesis (Fig. 1b). Loss of proteostasis activates the unfolded protein response, thereby sustaining chronic, tumor-promoting inflammation [12]. Mitochondrial dysfunction, through increased reactive oxygen species (ROS) production and reprogramming of the tricarboxylic acid cycle, reshapes neoplastic energy metabolism. Disrupted intercellular communication, mediated by senescence-associated secretory phenotype (SASP) factors, extracellular vesicles, and extracellular matrix remodeling, simultaneously promotes inflammation, reinforces senescence, reprograms metabolism, and facilitates immune evasion [13]. Immune escape, in turn, engages in complex crosstalk with chronic low-grade inflammation, microbiome dysbiosis, persistent senescence, and broader network remodeling. Collectively, these systemic, aging-driven alterations orchestrate cancer initiation and progression through parallel and sequential mechanisms, providing a robust theoretical basis for “one drug, two targets” therapeutic strategies.

### The paradox of aging and cancer: aging increases cancer risk, whereas cancer therapies accelerate aging

**Fig. 2:**
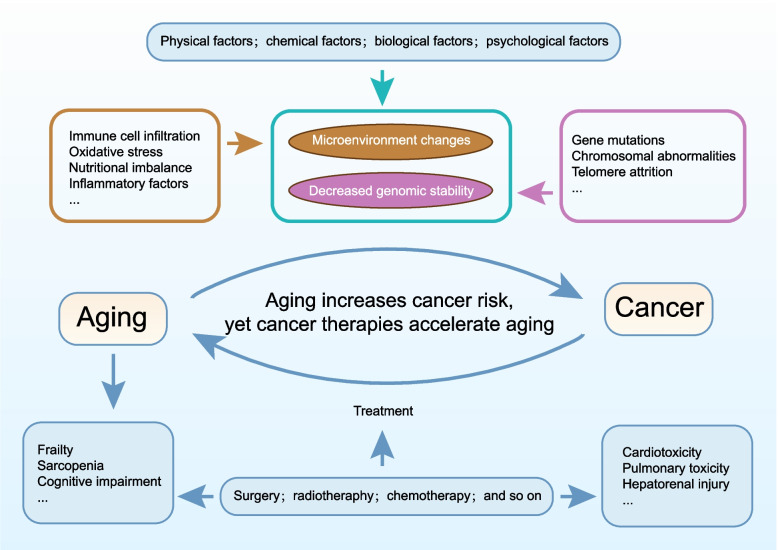
Cancer incidence is strongly age-dependent. Biological, physical, chemical, and psychosocial insults collectively drive organismal aging, manifesting as a progressive loss of genomic stability and systemic remodeling of the tissue microenvironment. Current antineoplastic modalities, while suppressing cancer growth, simultaneously induce therapy-induced senescence (TIS) and cumulative toxicity, thereby exacerbating aging-related comorbidities in older patients

The multilayered interaction model illustrated in Fig. 2 depicts the age-associated increase in cancer risk. With advancing age, progressive genomic erosion drives an exponential rise in mutational burden and chromosomal abnormalities, creating a permissive genetic substrate for malignant transformation. Concurrently, the tissue microenvironment undergoes systemic remodeling: oxygen tension and nutrient availability decline along increasingly steep gradients, extracellular matrix cross-linking and stiffness increase, and immune infiltration shifts from effective surveillance toward a chronic inflammatory phenotype [14]. Together with genomic instability, these microenvironmental alterations amplify clonal selection pressures and substantially elevate cancer incidence. A key contributing factor is the accumulation of senescent cells, which establish an immunosuppressive, tumor-promoting milieu through SASP-mediated release of inflammatory mediators such as interleukin (IL)−6 and IL-8, thereby facilitating tumor initiation and progression.

Conversely, malignant disease and its therapeutic interventions can accelerate organismal aging. Although chemotherapy, radiotherapy, and immunotherapy effectively suppress tumor growth and metastasis, they also inflict substantial off-target damage on normal tissues [15]. Cytotoxic agents induce DNA double-strand breaks, triggering cell-cycle arrest, apoptosis, mitochondrial dysfunction, and ROS surges that promote cellular senescence. Similarly, ionizing radiation causes persistent oxidative stress and genomic instability through overlapping mechanisms. Immune-based therapies reinvigorate anticancer immunity; however, sustained high-intensity activation may drive T-cell exhaustion, restrict memory T-cell diversity, and remodel immune regulatory networks, ultimately culminating in immunosenescence [16]. Collectively, these processes lead to therapy-induced senescence (TIS), which compromises quality of life and increases the risk of late toxicities and secondary complications [17].

## Synergistic pro-carcinogenic factors in aging

Given the higher cancer incidence and mortality observed in older adults, we focus on aging-associated hallmarks that synergistically promote tumor development. Given the higher incidence and mortality of cancer observed in older adults, we focus on aging-associated hallmarks that synergistically promote tumor development. By analyzing the hallmarks of aging and cancer, convergent characteristics between the two can be observed in terms of genetics, epigenetics, immunity, microbiota, and energy changes. We discuss the characteristics of aging and cancer in these five aspects and further analyze their molecular crosstalk patterns.

### Genomic instability

Genomic stability is fundamental to cellular homeostasis and serves to restrain both organismal aging and malignant transformation. Nuclear and mitochondrial DNA are continuously exposed to exogenous insults, such as ionizing radiation and chemical mutagens, as well as endogenous stressors including ROS and replication errors, resulting in single- and double-strand breaks, base lesions, and chromosomal translocations [18]. To counteract this damage, cells rely on a hierarchically organized DNA repair network encompassing base excision repair, nucleotide excision repair, mismatch repair, homologous recombination, non-homologous end joining, and mitochondria-specific mechanisms, together with chromatin remodeling and telomere maintenance pathways [19, 20]. Collectively, these systems preserve chromosomal integrity and transcriptional fidelity.

The efficiency of these repair mechanisms progressively declines with age, leading to an increasing imbalance between DNA damage accumulation and repair capacity [21]. Consequently, mutational burden, chromosomal rearrangements, and mitochondrial DNA deletions increase substantially, thereby amplifying genomic instability. On one hand, persistent genomic damage activates sustained DNA-damage responses (DDRs) that enforce cellular senescence, deplete stem cell pools, and impair tissue regeneration, accelerating systemic functional decline. On the other hand, accumulated mutations may confer selective growth advantages, promoting oncogene activation, inactivation of tumor suppressors, and the molecular evolution of malignant clones.

In malignant cells, key DNA repair pathways, including homologous recombination, non-homologous end joining, and mismatch repair, are frequently compromised, allowing DNA lesions to accumulate at an accelerated rate [22, 23]. Mutations or epigenetic silencing of genome guardian genes such as tumor protein p53 (*TP53)* and breast cancer susceptibility gene 1/2 (*BRCA1/2)* further weaken DDRs, increasing mutational load and driving rapid clonal diversification [24]. Recent studies have identified the zinc-finger protein zinc finger protein 451 (ZNF451) as a positive regulator of DNA repair efficiency through small ubiquitin-like modifier 2 (SUMO2)-mediated modification of ring finger protein 168 (RNF168); loss of ZNF451 reduces repair capacity and enhances chemosensitivity in cancer cells [25]. Thus, DNA repair deficiencies not only initiate malignant transformation but also shape tumor responsiveness to cytotoxic and targeted therapies.

Genomic instability in cancer manifests at both chromosomal and nucleotide levels. Macroscopic alterations include numerical aberrations (gains and losses) and structural rearrangements such as translocations, inversions, and amplifications, whereas microscopic alterations include single-base substitutions and small insertions or deletions [26, 27]. Together, these alterations confer clonal plasticity, enabling neoplastic cells to adapt to metabolic stress, immune surveillance, and therapeutic pressure. Chronic myeloid leukemia exemplifies this principle: its characteristic Philadelphia chromosome, t(9;22)(q34;q11), generates the constitutively active BCR::ABL1 tyrosine kinase, driving malignant transformation of hematopoietic stem cells (HSCs) [28]. Similarly, defective mismatch repair underlies microsatellite instability in colorectal and endometrial cancers, resulting in frameshift mutations and neoantigen accumulation that reshape tumor immunophenotype and influence clinical outcomes [29, 30]. Recurrent mutations in *TP53*, kirsten rat sarcoma viral oncogene homolog (*KRAS)*, and *MYC* further disrupt cell-cycle regulation, apoptosis, and proliferative signaling while also serving as biomarkers for molecular stratification and therapeutic targeting [31].

Clonal expansion driven by genomic instability markedly increases intratumoral heterogeneity, endowing cancer populations with extensive genetic and phenotypic diversity that facilitates adaptation to diverse environmental pressures [32]. During tumor evolution, subclones harboring advantageous mutations progressively outcompete neighboring populations through clonal selection, ultimately reshaping the tumor ecosystem [33]. This dynamic process underlies increasing biological complexity and represents a central mechanism of therapeutic resistance. For example, in non-small-cell lung cancer (NSCLC), tobacco-associated genomic injury is frequently accompanied by copy number alterations that synergistically enhance proliferative capacity and confer resistance to chemotherapy [34].

Collectively, genomic instability represents a central mechanistic link between aging and cancer. With advancing age, the gradual accumulation of somatic mutations increases mutational burden, compromises physiological function, and creates a permissive environment for carcinogenesis [35]. Declining DDR capacity further exacerbates genomic rearrangements and point mutations. Clonal hematopoiesis provides a clear illustration: HSCs harboring driver mutations in DNA methyltransferase 3 alpha (*DNMT3A)*, ten–eleven translocation 2 (*TET2)*, or additional sex combs-like 1 (*ASXL1)* gain proliferative advantages and increase the risk of hematologic malignancies in older individuals [36]. Likewise, *TP53* mutations accumulate in aged tissues, generating p53 variants with impaired target recognition, weakened checkpoint control, and unchecked proliferation [37]. Whole-genome sequencing studies have revealed that normal esophageal epithelium can harbor extensive somatic mutations and clonal expansions by midlife, with *TP53* mutations enriched in esophageal carcinoma [38]. In a study based on biopsies of normal human eyelid epidermis, normal cells were found to harbor 3,760 somatic mutations, which are commonly observed in skin cancers [39]. Notably, one-quarter of the cells in the biopsies harbored *NOTCH* mutations, a key oncogenic driver, indicating a growth advantage [40, 41]. However, unlike cancer cells, the range of clone sizes remained limited. These findings suggest that age-associated clonal mutations are widespread and may remain benign until environmental stressors or additional insults drive the transition toward overt malignancy.

### Epigenomic changes

Epigenetic remodeling represents another central hallmark of aging and encompasses spatiotemporal reprogramming of DNA methylation, imbalances in histone post-translational modifications, alterations in the RNA epitranscriptome, and widespread disruption of noncoding RNA (ncRNA) regulatory networks.

With advancing age, global DNA methylation progressively declines, whereas focal hypermethylation emerges at promoter CpG islands [42]. Juan et al. measured DNA methylation levels across multiple mouse strains and observed substantial interstrain variability in baseline methylomes; however, age-associated methylation changes were highly consistent across strains and were similarly observed in humans [43]. Himani et al. further examined the relationship between DNA methylation entropy (quantified using the Jensen–Shannon divergence) and chronological age across seven tissues, demonstrating that age-related methylation drift and increased entropy arise largely from cumulative stem cell replication in adult tissues [44]. Together, these findings underscore the biological relevance of DNA methylation–based clocks. DNA methylation clocks, derived from specific methylation signatures, serve as robust biomarkers of biological aging, with the capacity to predict mortality risk and to evaluate interventions with potential to delay aging and extend lifespan [45, 46].

Mislocalization of histone marks such as H3K4me3, H3K9me3, and H3K27ac alters chromatin accessibility and transcriptional regulation. Concurrently, age-associated heterochromatin reorganization mediated by four ATP-dependent chromatin-remodeling complexes SWItch/sucrose non-fermentable (SWI/SNF), imitation switch (ISWI), chromodomain helicase DNA-binding (CHD), and inositol-requiring 80 (INO80) further amplifies genomic instability [11, 47]. The RNA epigenetic landscape also undergoes age-related drift; modifications including m6A, m5C, and A-to-I editing modulate mRNA stability and translational efficiency [48, 49]. ncRNAs fine-tune gene expression programs involved in aging. For example, microRNA (miR)−455-3p extends lifespan in mice, whereas mesenchymal stem cell–derived small extracellular vesicles enriched in miR-146a markedly attenuate vascular endothelial senescence [50].

Epigenetic alterations thus constitute a critical mechanistic link between aging and cancer. As epigenetic regulation becomes progressively dysregulated with age, aberrant gene expression patterns emerge that compromise cellular homeostasis and simultaneously facilitate malignant transformation, reinforcing the intimate connection between aging and cancer at the epigenetic level.

Epigenetic reprogramming underlies cancer cell plasticity and adaptability [51]. Global DNA hypomethylation promotes chromosomal instability, whereas focal promoter hypermethylation silences tumor-suppressor genes [52]. Hypermethylation of CpG islands within tumor-suppressor gene promoters enforces transcriptional repression. Notably, methyl-CpG–binding domain protein 2 forms nuclear condensates in multiple cancer types, where it recruits the nucleosome remodeling and deacetylation (NuRD) complex to specific genomic loci, thereby mediating gene silencing and promoting tumor progression. Nadezhda et al. performed chromatin accessibility and transcriptomic analyses of 225 samples across 11 cancer types, revealing marked intertumoral differences in chromatin accessibility; in breast cancer, these alterations were closely associated with *TP53* mutations [53].

Histone modifications further remodel the chromatin landscape in cancer. The histone regulator lysine demethylase 1B (KDM1B) binds promoter regions of stemness-associated genes in cancer stem cells (CSCs), modulating chromatin accessibility and facilitating tumor progression [54]. Using cellular and murine cervical cancer models, Li et al. demonstrated that the histone methyltransferase (HMT) suppressor of variegation 3–9 homolog 1 (SUV39H1) increases H3K9me3 levels and induces DNMT3A expression, thereby suppressing immunosuppressive factor expression through DNA methylation [55]. Additionally, transient disruption of Polycomb group complexes has been shown to irreversibly drive Drosophila cells toward malignant transformation [56]. Dysregulation of RNA epigenetic mechanisms, particularly the m6A machinery, is also strongly implicated in cancer pathogenesis [57]. In acute myeloid leukemia (AML), methyltransferase-like 3 (METTL3) overexpression maintains leukemic stem cells in an undifferentiated state, whereas fat mass and obesity-associated protein (FTO) and AlkB homolog 5 (ALKBH5) inhibit apoptosis, promote proliferation, and confer drug resistance [58, 59]. MicroRNA networks function as both oncogenic drivers and minimally invasive biomarkers; for example, imbalance in the miR-21/miR-122 axis suppresses programmed cell death protein 4 (*PDCD4)*, accelerating hepatocellular proliferation [60].

In summary, the epigenomes of aging and cancer converge through shared destabilization. Global DNA hypomethylation coupled with focal hypermethylation characterizes both the aging epigenetic clock and early oncogenesis [61]. Age-associated expansion of *DNMT3A*-mutant clonal hematopoiesis in the bone marrow predisposes individuals to AML and myelodysplastic syndromes (MDS) [62, 63]. MicroRNAs exhibit parallel alterations, with elevated miR-23a and miR-155 linking cellular senescence to carcinogenesis [64, 65]. Moreover, METTL3-driven m6A modification amplifies SASP output and sustains leukemic stemness in AML [66, 67].

### Immunological dysregulation and chronic inflammation

With advancing age, the immune system progressively acquires features of immunosenescence, leading to impaired clearance of mutated or transformed cells and creating opportunities for cancer cells to evade immune surveillance. Concurrently, age-related chronic low-grade inflammation, termed inflammaging, induces persistent alterations in the tissue microenvironment that facilitate tumor cell proliferation, angiogenesis, and metastatic dissemination.

**Fig. 3:**
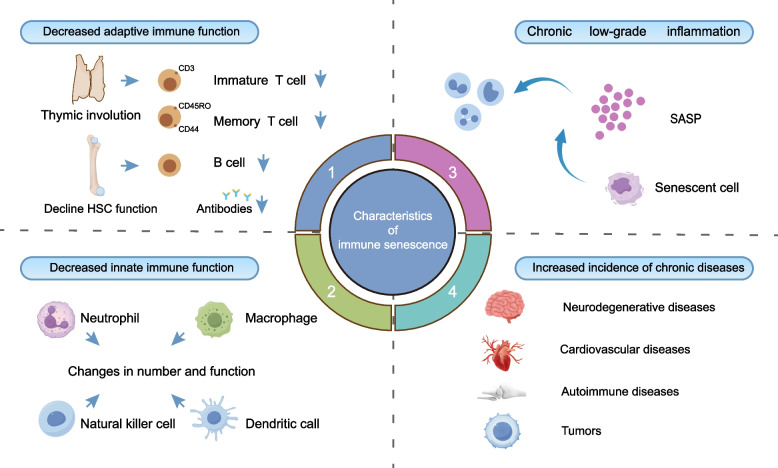
Hallmarks of immunosenescence

Immunosenescence is a complex biological process characterized by the gradual decline of immune competence with age. It involves a series of dynamic, progressive, and largely irreversible immune-remodeling events that unfold across the life span (Fig. 3) [68].

The adaptive immune system is particularly affected during aging. Thymic involution leads to a marked reduction in naïve T-cell output, whereas memory T cells accumulate as a compensatory response, resulting in a narrowed T-cell receptor repertoire [69]. Concurrently, the differentiation potential and clonal expansion capacity of B lymphocytes in the bone marrow decline, whereas the proportion of proinflammatory B-cell subsets in peripheral blood increases [70]. Together, these adaptive immune alterations substantially impair the ability of older individuals to recognize and eliminate novel or recurrent pathogens, thereby increasing susceptibility to infection. T-cell senescence plays a central role in this process. In humans, cluster of differentiation (CD)4^+^ and CD8^+^ T cells exhibit differential susceptibility to senescence, with CD8^+^ T cells acquiring an immunosenescent phenotype more rapidly than their CD4^+^ counterparts [71].

In parallel, the innate immune system is also compromised with age. Neutrophils exhibit defects in chemotaxis and phagocytic killing, whereas dendritic cells show pronounced impairments in antigen capture, processing, and migration to secondary lymphoid organs [72]. Moreover, macrophages display disrupted expression of circadian rhythm–related genes, further impairing innate immune regulation [68]. Collectively, these innate immune deficits compromise early pathogen recognition and rapid clearance, thereby creating opportunities for latent infections and opportunistic microbes to establish themselves.

Inflammaging is not merely a consequence of immunosenescence but also a driving force that accelerates its progression [73]. This persistent inflammatory state is sustained by the SASP and is further amplified by the accumulation of damage-associated and pathogen-associated molecular patterns [74]. Together, immune decline and inflammaging disrupt physiological homeostasis and establish a bidirectional axis that underlies the development of major chronic diseases, including cardiovascular disorders, neurodegeneration, autoimmunity, and cancer [75].

During early carcinogenesis, the immune system can eliminate nascent malignant clones through immunosurveillance. However, as tumors progress, cancer cells deploy multiple immune-evasion strategies that enable unchecked proliferation and subsequent dissemination [76]. These mechanisms can be broadly classified into three interrelated categories. (1) Immunogenic attenuation occurs when cancer cells downregulate neoantigen expression and major histocompatibility complex (MHC) molecules, thereby reducing their visibility to immune effectors [77, 78]. (2) Establishment of the tumor immunosuppressive microenvironment (TIME) is driven by tumor-derived immunosuppressive cytokines that recruit regulatory immune populations; tumor-associated macrophages (TAMs) further reinforce the TIME through inflammatory signaling and polarization [79, 80]. (3) Suppressive signaling activation involves the chronic upregulation of immune checkpoint pathways such as programmed cell death protein 1 (PD-1)/programmed death-ligand 1 (PD-L1) and cytotoxic T-lymphocyte–associated protein 4 (CTLA-4), which cooperate with *MYC*- and *KRAS*-driven transcriptional reprogramming to accelerate tumor growth and intensify immunosuppression [79, 80].

Immunosuppressive cells within the tumor microenvironment (TME), including myeloid-derived suppressor cells (MDSCs), regulatory T cells (Tregs), and TAMs, act in concert with tumor cells to secrete immunosuppressive factors, thereby inhibiting antitumor immune responses [77]. Metabolic alterations within the TME further compromise immune cell function. Engagement of PD-1 with its ligand PD-L1 suppresses T-cell activation, proliferation, and cytokine production, ultimately leading to T-cell exhaustion [81]. Tumor cells frequently overexpress PD-L1, impairing the function of tumor-infiltrating lymphocytes and facilitating immune evasion. Tumor cells also upregulate CTLA-4, which binds CD80/CD86 to inhibit T-cell activation and suppress effector function [82].

Within this intricate immunoregulatory network, CD8^+^ T cells represent a critical nexus linking aging-related immune dysfunction to cancer progression. Aging disrupts the activation and differentiation of cytotoxic CD8^+^ T cells within tumors, resulting in the loss of tumor antigen–specific CD8^+^ T cells (CD8 TCR-T cells) accompanied by downregulation of Epas1, a key regulator within the hypoxia-inducible factor family. This phenomenon has been demonstrated in adoptive T-cell therapy models: transfer of CD8 TCR-T cells from young mice into aged, tumor-bearing hosts significantly delayed tumor progression and restored responsiveness to PD-1 immune checkpoint blockade [83]. Furthermore, exogenous Epas1 supplementation ameliorated functional deficits in senescent CD8 TCR-T cells in aged tumor-bearing mouse models, enhancing tumor infiltration, persistence, and antitumor activity [84].

Aged CD8^+^ T cells exhibit proliferative defects and increased susceptibility to genotoxic stress, rendering them prone to terminal exhaustion within the TME [85, 86]. T-cell exhaustion, driven by chronic antigen exposure (as observed in persistent viral infections and cancer) is characterized by a progressive loss of effector functions, sustained expression of inhibitory receptors, and persistent activity of the transcription factor thymocyte selection-associated high mobility group box protein TOX (TOX) [87]. The difficulty in durably reversing T-cell exhaustion remains a major therapeutic challenge in both oncology and chronic infectious diseases.

In summary, the markedly reduced cytotoxic capacity of senescent CD8^+^ T cells, together with the accumulation of immunosuppressive cells and immune checkpoint signaling within the TIME, amplifies its immunosuppressive state. This cascade weakens the magnitude and durability of antitumor immune responses, ultimately establishing an immunologically permissive microenvironment that facilitates tumor initiation, progression, and metastasis.

### Dysbiosis

With advancing age, profound alterations occur in the composition and function of the gut microbiome. This age-associated dysbiosis can exacerbate systemic inflammation and impair immune surveillance, thereby accelerating biological aging and increasing susceptibility to cancer.

The commensal microbiota, anchored within the intestinal ecosystem, serves as a key regulator of systemic homeostasis and aging. Its composition and metabolic profile undergo profound age-associated remodeling [88]. In older adults, microbial diversity declines markedly, with the abundance of beneficial taxa such as *Bifidobacterium* spp. and *Firmicutes* decreasing, whereas that of potentially pathogenic taxa, including Enterobacteriaceae and Streptococcus spp., increases [89]. This dysbiotic shift promotes chronic low-grade intestinal inflammation, impairs mucosal immune surveillance, and weakens systemic immune competence. Moreover, loss of butyrate-producing bacteria reduces short-chain fatty acid synthesis, disrupts epithelial tight-junction integrity, and exacerbates gut barrier permeability [90].

Genetic and functional variability within the microbiome strongly influences carcinogenesis, tumor progression, and therapeutic response, with the gut microbiota playing a central role in these processes. The existence of intratumoral microbiota (microorganisms residing within tumor tissues) has now been firmly established [91, 92]. Early studies showed that intratumoral microbes and their metabolites can promote oncogenesis by inducing direct host DNA damage or by shaping the TME. More recent mechanistic investigations have demonstrated that across diverse cancer models, these microbes or their metabolites can exert context-dependent effects that are either tumor-promoting or tumor-suppressive [93]. For example, in pancreatic cancer, specific intratumoral taxa are associated with prolonged patient survival, suggesting potential antitumor functions [94]. Conversely, Lactobacillus iners has been shown to promote chemoradioresistance in cervical cancer and is associated with reduced patient survival [95].

The interplay between aging and malignancy is complex, with the gut microbiome emerging as a key, multilayered regulator along this continuum. With advancing age, the intestinal microbiota undergoes pronounced shifts in both community composition and metabolic capacity. This dysbiotic state compromises epithelial tight-junction integrity, weakens intestinal barrier function, and promotes chronic low-grade inflammation, thereby creating a protumorigenic microenvironment [96].

Immune senescence further exacerbates this risk. T-cell function declines with age, characterized by reduced cytotoxic capacity, altered cytokine secretion, and senescent phenotype accumulation, particularly in middle-aged and older individuals. Notably, reduced microbial diversity correlates with increased expression of T-cell senescence markers in adults aged ≥ 45 years, highlighting an age-dependent gut–immune association [97]. Studies of long-lived cohorts integrating metagenomic and cytokine profiling have revealed a close relationship between microbiota alterations, disrupted metabolic pathways, and chronic inflammation. This association promotes immunosenescence and may establish a self-reinforcing cycle that disrupts gut homeostasis and increases susceptibility to cancer and other age-related chronic diseases [98, 99].

### Nutrient-sensing dysregulation

Dysregulated nutrient sensing is recognized as a hallmark of aging and is characterized by systemic disruption of the nutrient-sensing network centered on insulin/insulin like growth factor 1 (IGF-1), mechanistic target of rapamycin (mTOR), adenosine monophosphate-activated protein kinase (AMPK), and sirtuins (SIRT) [11]. Through tightly interconnected signaling cascades, this network maintains anabolic–catabolic balance and coordinates metabolic homeostasis of glucose, amino acids, and lipids [100].

Systemic glucose regulation progressively deteriorates with age and is accompanied by mitochondrial dysfunction. Expression of key glycolytic enzymes, such as hexokinase 2 (HK2) and lactate dehydrogenase A (LDHA), becomes upregulated, driving aerobic glycolysis (the Warburg effect) and lactate accumulation, which in turn induces metabolic acidosis and cellular injury [101, 102]. Pyruvate dehydrogenase kinase 4 (PDK4) is also upregulated during aging, further promoting the Warburg effect and lactate production [103]. Intersecting signaling axes involving AMPK, SIRT, and nuclear factor kappa-B (NF-κB) coordinate metabolic reprogramming in senescent cells, precisely modulating responses to energetic stress and inflammatory outputs [104].

Amino acid metabolism also undergoes pronounced age-related alterations. Systemic levels of glutamine and τ-methylhistidine increase, whereas those of arginine and β-hydroxy-β-methylbutyrate decline. Concurrently, glutamine and leucine clearance rates increase, whereas arginine clearance rate decreases [105]. These metabolic shifts may reflect dysregulated mTOR complex 1 (mTORC1) activity, which governs amino acid transport and catabolism through phosphorylation-dependent signaling cascades [106]. Additionally, the insulin/IGF-1 signaling pathway plays a critical role in modulating the SIRT1–p53 axis, and sustained activation of this pathway has been implicated in the promotion of aging [107].

In lipid metabolism, aging is characterized by excessive lipid accumulation driven by increased exogenous uptake, upregulated de novo synthesis, and reduced catabolic efficiency [108]. In cholesterol homeostasis, intracellular cholesterol levels increase during aging as a result of both enhanced uptake and increased de novo synthesis [109]. The cholesterol transporter ATP-binding cassette A1 (ABCA1) has been shown to facilitate cholesterol import into lysosomes, leading to lysosomal cholesterol accumulation [110]. This accumulation promotes the formation of cholesterol-enriched microdomains that are rich in mTORC1 scaffold complexes, thereby sustaining mTORC1 activity and enhancing SASP expression.

Metabolic reprogramming represents a key shared feature of aging and cancer. During aging, cellular metabolism progressively shifts toward reduced energetic efficiency, leading to functional decline and diminished tissue regenerative capacity. Conversely, during tumor initiation and progression, cancer cells actively rewire metabolic pathways to meet the increased bioenergetic and biosynthetic demands of rapid proliferation.

Cancer metabolic reprogramming refers to the extensive remodeling of nutrient utilization undertaken by malignant cells to sustain uncontrolled growth. This process involves altered uptake, utilization, and redistribution of glucose, glutamine, fatty acids, and serine/glycine, resulting in metabolic profiles and adaptive capacities that are distinct from those of normal somatic cells [111].

Even in the presence of sufficient oxygen, cancer cells preferentially rely on glycolysis rather than the more energy-efficient oxidative phosphorylation to generate ATP, a phenomenon known as aerobic glycolysis or the Warburg effect [112]. The molecular architecture underlying this metabolic shift is complex and is driven by the convergent effects of oncogene activation, tumor-suppressor function loss, mitochondrial DNA mutations, and dynamic microenvironmental cues [113]. For instance, overexpression of the *MYC* oncogene upregulates glycolysis-associated genes, including those encoding glucose transporters, thereby enhancing glycolytic flux [114]. Similarly, hypoxia-inducible factor, activated under hypoxic conditions, induces the expression of key glycolytic enzymes such as HK2, phosphoglycerate kinase 1, and LDHA [115]. Collectively, these adaptations enable cancer cells to rapidly generate ATP and metabolic intermediates to support accelerated proliferation.

Cancer cells also exhibit pronounced glutamine dependence, with glutamine uptake often exceeding biosynthetic demand, resulting in net depletion. In vitro depletion of exogenous glutamine induces growth arrest or apoptosis, underscoring this critical metabolic dependency [116, 117]. Glutamine reprogramming is tightly coupled to oncogenic signaling pathways. Under nutrient-deprived conditions, mTORC1 promotes lipophagy and suppresses cholesterol synthesis, whereas activation of the phosphatidylinositol 3-kinase (PI3K)/protein kinase B (PKB/AKT) pathway upregulates the glutamine transporter solute carrier family 1 member 5 (SLC1A5), thereby enhancing glutamine uptake and utilization [118, 119]. Glutamine metabolism is particularly important in CSCs. Within CSCs and the TME, glutamine metabolism maintains redox homeostasis and contributes to epigenetic regulation, thereby supporting stemness and survival [120]. Moreover, glutamine plays a central role in cancer initiation, progression, metastasis, and therapeutic resistance. In a mouse model of bladder cancer, upregulation of SLC6A14 enhanced glutamine catabolism, promoting cancer cell proliferation, migration, and invasion [121]. Similar observations have been reported in NSCLC [122]. Additionally, enhanced glutamine metabolism has been shown to confer resistance to epidermal growth factor receptor (EGFR) inhibitors in NSCLC cells [123].

Lipid metabolic reprogramming is equally critical to cancer progression and involves increased lipid uptake, enhanced de novo lipogenesis, fatty acid oxidation, and lipid storage [124]. Fatty acid synthase (FASN), the rate-limiting enzyme in this pathway, drives de novo lipid biosynthesis, supplying membrane components, signaling intermediates, and antiapoptotic factors essential for malignant proliferation and survival [125]. This metabolic rewiring is tightly regulated by the mTORC1, PI3K/AKT, and AMPK signaling axes. Upon activation, these pathways coordinately promote de novo fatty acid synthesis and cellular lipid uptake [126]. Consequently, targeted inhibition of these signaling networks can induce cancer cell apoptosis. In pancreatic ductal adenocarcinoma, the KRAS–mitogen-activated protein kinase (MAPK) signaling pathway serves as a major driver of de novo lipid metabolism. Combined targeting of PIKfyve (a lipid kinase essential for lysosomal function) and the KRAS–MAPK pathway has been shown to substantially reduce tumor burden in multiple preclinical human and mouse models [127].

Although aging and cancer display distinct metabolic phenotypes, their shared molecular landscape reveals deep interconnections, indicating that age-related metabolic reprogramming may precondition a biochemical milieu conducive to malignant transformation. The following sections examine this overlap across the three principal nutrient classes and their key metabolic derivatives.

Carbohydrates: Both senescent and malignant cells exhibit substantially increased glycolytic activity. With advancing age, glycolysis is upregulated, and a greater proportion of glucose-derived carbon is redirected into the tricarboxylic acid cycle, rendering cellular energy metabolism increasingly glycolysis dependent. This shift mirrors the Warburg phenotype and supplies cancer cells with rapid ATP production and essential biosynthetic precursors [128].

Amino acids: Dysregulated amino acid metabolism, particularly glutamine handling, represents another shared feature. Systemic glutamine depletion has been documented in multiple age-related disorders [129]. In aged rats, elevated glutamine synthetase activity reflects increased metabolic demand, closely resembling the glutamine-addicted state characteristic of many malignancies [130].

Lipids: Lipid metabolism is likewise reprogrammed in both senescence and cancer. In addition to increased lipid uptake, de novo fatty acid synthesis constitutes a major lipid source [109]. During aging, FASN expression increases markedly, and its inhibition attenuates senescence in both murine and human cells. In malignancies, elevated FASN activity supports tumor growth and contributes to metastasis and therapeutic resistance [109, 131].

In addition to these core nutrient axes, further points of convergence arise in mitochondrial dynamics, lactate turnover, and the folate cycle [132]. Collectively, this metabolic convergence between aging and malignancy provides a robust theoretical framework for elucidating carcinogenic mechanisms and for developing integrated strategies that simultaneously modulate aging and target cancer.

## Antagonistic tumor-suppressive factors in aging

The preceding sections have focused on aging-associated hallmarks that synergistically promote cancer development. However, not all aged individuals develop cancer, and it is equally important to recognize that aging also involves antagonistic factors that exert tumor-suppressive effectss. From the perspective of hallmarks, antagonistic molecular signatures exist between aging and cancer, most notably those related to telomere integrity and the cellular stemness maintenance. Specifically, during aging, telomeres gradually shorten and cellular stemness is progressively lost, leading to declined cellular function or cell death. In contrast, cancer cells can evade such outcomes through multiple mechanisms to sustain cell survival, ultimately posing a threat to organismal health.

### Telomere attrition

Telomere integrity is essential for sustained cellular proliferation, and telomere dysfunction is closely linked to both aging and cancer. During aging, progressive telomere shortening ultimately triggers cellular senescence or apoptosis, thereby limiting tissue regenerative capacity. Conversely, cancer cells frequently maintain telomere length through aberrant mechanisms, most notably telomerase activation, which enables unlimited proliferative potential.

Telomeres consist of tandem TTAGGG repeats bound by the shelterin protein complex [133]. In eukaryotic cells, DNA polymerase is unable to fully replicate telomeric regions, resulting in progressive telomere shortening with each cell division. Telomerase, a ribonucleoprotein complex, counteracts this attrition by adding repetitive DNA sequences to telomeres, thereby maintaining telomere length [134]. In most somatic cells, telomerase activity is minimal; consequently, telomeres progressively shorten with successive divisions until a critical length is reached. At this point, a DDR is activated, engaging the p53/p21 and p16^INK4a^ pathways and ultimately inducing replicative senescence [135, 136]. Telomere length declines with increasing chronological age, as confirmed by recent studies, which further indicate that this process follows a nonlinear trajectory [137]. Beyond the cellular level, telomere attrition exerts systemic effects across tissues and organs, contributing to skin aging, neurodegeneration, cardiovascular disease, and diabetes mellitus [138].

Telomere shortening is a common precancerous feature observed during early tumorigenesis [139, 140]. At this stage, telomere shortening–induced cellular senescence acts as a protective mechanism that limits genomic instability and suppresses tumor growth. However, excessive telomere erosion can trigger widespread apoptosis, a phenomenon termed telomere-induced crisis [9]. A small subset of cells escapes this barrier by acquiring telomere maintenance mechanisms (TMMs). Approximately 80%–85% of human cancers reactivate telomerase through mechanisms such as human telomerase reverse transcriptase (*hTERT)* promoter mutations, *TERT* rearrangements, viral insertion, or Fe^3+^-dependent transcriptional upregulation, leading to increased *TERT* expression and conferring a proliferative advantage on malignant stem cells [141, 142]. In MDS and myelofibrosis (MF), neoplastic HSCs harbor critically short telomeres but display elevated telomerase activity, reflecting a compensatory response that supports clonal persistence and disease progression [143].

Additionally, approximately 10%–15% of cancers maintain telomere length through the telomerase-independent alternative lengthening of telomeres (ALT) pathway, a process driven primarily by RAD52-mediated break-induced DNA replication [144]. ALT is characterized by pronounced telomere length heterogeneity and extensive chromosomal instability [145]. This mechanism occurs in a tissue-specific spectrum of malignancies and is particularly prevalent in tumors of neuroepithelial and mesenchymal origin [146, 147]. Although only a minority of tumors rely on ALT for telomere maintenance, ALT-positive cancers often exhibit higher malignant potential and enhanced migratory capacity compared with telomerase-positive tumors [148]. Moreover, tumors can acquire ALT as an adaptive response following anti-telomerase therapy, thereby facilitating disease relapse.

Telomere vesicle transfer represents a third, more recently described TMM that is independent of telomerase. Through this process, naïve T cells acquire telomeric fragments from antigen-presenting cells, thereby extending their own telomeres [149, 150]. Although this mechanism has not yet been demonstrated in cancer, intercellular telomere trafficking constitutes a compelling translational avenue for modulating telomere dynamics within the TME.

### Stem cell exhaustion

During aging, the progressive depletion of tissue-resident stem cells leads to diminished regenerative capacity and subsequent deterioration of organ function. Conversely, during tumor initiation and progression, a subset of cancer cells acquires stem cell–like properties that support tumorigenesis and disease persistence.

Stem cell competence declines with age and is accompanied by reduced cellular plasticity. Aged HSCs exhibit impaired bone marrow homing and, during long-term co-culture with stromal cells, demonstrate markedly reduced seeding efficiency and delayed proliferative responses compared with their younger counterparts [151]. This attrition contributes to the increased susceptibility of older individuals to sarcopenia, osteoporosis, anemia, and neurodegenerative disorders. In skeletal muscle, aging and chronic myopathies disrupt satellite cell homeostasis, leading to progressive depletion of the muscle stem cell pool. This loss drives a sustained decline in regenerative capacity, ultimately resulting in irreversible reductions in muscle mass and contractile strength [152]. Within the hematopoietic system, age-related impairment of regenerative capacity limits adaptive immune cell output and increases the risk of anemia and myeloid malignancies [153].

Cellular plasticity underlies intratumoral heterogeneity, enabling cancer cells to dynamically transition among distinct differentiation states [154]. This capacity is sustained by a tightly coordinated network integrating genetic alterations, transcriptional reprogramming, signaling pathway rewiring, and microenvironmental cues, thereby driving continuous tumor evolution [155–157]. CSCs, characterized by high plasticity, self-renewal, and multilineage differentiation potential, constitute a critical subpopulation that adapts to microenvironmental stress and promotes metastasis and disease relapse. Epithelial–mesenchymal transition (EMT) and its reverse process, mesenchymal–epithelial transition, exemplify these dynamic and reversible state transitions [158].

Malignant cells acquire plasticity through dedifferentiation, impaired differentiation, and transdifferentiation, processes that are frequently accompanied by dysregulated transcription factor activity and genomic aberrations [10]. Targeting these master regulatory programs offers a strategy to disrupt plasticity at its source and redirect malignant phenotypes. Acute promyelocytic leukemia provides a paradigm for the success of differentiation therapy: treatment with all-trans retinoic acid and arsenic trioxide, with or without chemotherapy, now achieves cure rates exceeding 90%. Furthermore, inflammatory signaling and the TIME can actively reinforce cellular plasticity, indicating that neutralization of inflammatory mediators or the strategic application of immunotherapies may further enable reversal of plastic states [159].

## Aging hallmarks with dual regulatory effects on cancer

The hallmarks of aging include not only tumor-promoting and tumor-suppressive factors but also features that exert dual regulatory effects on cancer. Among these, cellular senescence is particularly prominent: depending on microenvironmental context, senescent cells can exert opposing influences at different stages of tumor development. Notably, autophagy also displays bidirectional roles in cancer regulation and, in parallel, exerts dual effects on the aging process itself.

### Cellular senescence and TME

Cellular senescence is a stable state of growth arrest that cells enter in response to diverse forms of stress or damage. This process was initially recognized as a critical tumor-suppressive mechanism, preventing the proliferation of damaged cells and thereby limiting tumor initiation and early progression. However, with advancing age, the progressive accumulation of senescent cells contributes to systemic aging and promotes the development of age-related diseases.

The Hayflick limit exemplifies replicative exhaustion as a trigger of cellular senescence and represents a fundamental barrier to malignant transformation [160, 161]. Telomere attrition, DNA damage, ROS accumulation, and oncogene activation converge to impose cell-cycle arrest, thereby restricting early clonal expansion [162]. Nevertheless, the SASP exerts context-dependent effects. In the acute setting, SASP factors recruit immune cells to eliminate premalignant clones. Conversely, chronic secretion of mediators such as IL-6 and C-X-C motif chemokine ligand (CXCL1) sustains a proinflammatory microenvironment that delivers growth and survival signals to residual cancer cells, thereby facilitating tumor progression [163].

A similar duality is observed in TIS. Although TIS enforces growth arrest in tumor cells, the accompanying SASP can profoundly remodel the TME. This remodeling transforms the TME into a permissive niche that promotes immune evasion, therapeutic resistance, and metastatic relapse.

Through coordinated interactions between the SASP and TME, senescent cells impair natural killer (NK) and CD8^+^ T-cell function; upregulate PD-L1; and recruit MDSCs, Tregs, and TAMs, thereby establishing a strongly immunosuppressive barrier [76, 77]. Senescence has also been linked to drug resistance. In hepatocellular carcinoma, a SASP-associated p16/IL-6 axis drives acquired resistance to sorafenib following chronic exposure, whereas IL-6 blockade restores drug sensitivity [164]. Additionally, SASP-derived cytokines induce the expression of efflux transporters such as P-glycoprotein within the TME, reducing intracellular drug accumulation and contributing to chemoresistance [165]. Among SASP components, vascular endothelial growth factor and epidermal growth factor further promote tumor cell proliferation, angiogenesis, and EMT, thereby facilitating metastasis [163].

Reactivation of senescent cancer cells represents a critical mechanism underlying disease relapse. Although chemotherapy and radiotherapy can induce senescence in malignant cells, accumulating evidence indicates that under permissive conditions, these cells may re-enter the cell cycle, upregulate stemness-associated programs, and drive tumor recurrence and invasion [166].

### Autophagy dysfunction

Autophagy exerts dual regulatory roles in both aging and cancer. By eliminating damaged cellular components, autophagy delays aging and suppresses early tumorigenesis. However, with advancing age, declining autophagic activity accelerates organismal aging, whereas in established tumors, cancer cells can co-opt autophagy to support survival, growth, and therapy resistance.

**Fig. 4:**
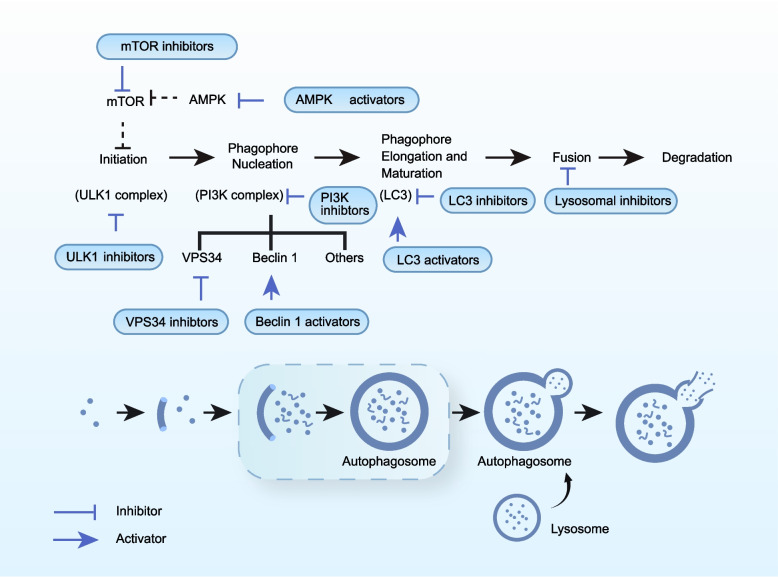
Autophagic flux and druggable nodes in cancer therapy [169, 170]. Macroautophagy proceeds through five mechanistically distinct phases: (i) initiation, (ii) phagophore nucleation, (iii) phagophore elongation and autophagosome maturation, (iv) autophagosome–lysosome fusion, and (v) lysosomal degradation of autophagic cargo. Pharmacologic interventions have been developed for each stage, yielding a range of targeted agents, including mTOR inhibitors, AMPK activators, ULK1 inhibitors, VPS34 inhibitors, Beclin-1 activators, and lysosomal inhibitors

Autophagy is an evolutionarily conserved lysosomal degradation pathway in eukaryotic cells (Fig. 4) [167]. Based on the route by which substrates are delivered to the lysosome, three forms are recognized: macroautophagy, microautophagy, and chaperone-mediated autophagy (CMA), with macroautophagy being the most extensively characterized. During aging, macroautophagic flux declines globally, primarily due to transcriptional downregulation of autophagy-related genes (ATGs) [168]. This age-associated impairment is now regarded as a central node accelerating functional decline.

Experimental evidence across species supports a protective role for autophagy in aging. In *Drosophila melanogaster*, reduced ATG expression shortens lifespan, whereas ATG8 overexpression extends longevity [168]. Similar effects have been observed in mammals. Insup et al. demonstrated that autophagy inhibition promotes microglial senescence and exacerbates neural damage in murine models of Alzheimer’s disease [171]. Genetic or pharmacologic activation of macroautophagy restores self-renewal and differentiation capacity in aged HSCs [172]. In vitro studies further indicate that glutamine suppresses glycolysis and reduces AMPK lactylation, thereby enhancing autophagy and limiting senescence in intervertebral disc cells [173]. γ-Mangosteen, a natural autophagy agonist, prevents oxidative stress–induced senescence in dermal fibroblasts both in vitro and in vivo [174]. Population-based studies have shown that peripheral blood mononuclear cells from centenarians and their offspring exhibit significantly elevated autophagic flux [175, 176]. Conversely, increased basal autophagy has also been observed in older individuals at high risk of diabetes, suggesting a compensatory response to accumulated cellular damage [177].

In addition to macroautophagy, CMA has emerged as an important regulator of aging. CMA activity declines with age across multiple tissues, and its impairment can recapitulate aging phenotypes. Conversely, CMA activation in mice prevents age-related disorders, including Alzheimer’s disease, retinal degeneration, and atherosclerosis [178, 179].

Although substantial evidence supports autophagy activation as a longevity-promoting mechanism, emerging data indicate that autophagy can also promote senescence in a context-dependent manner [180, 181]. The “autophagy–stress intensity” model proposes that mild or transient autophagy delays aging by removing damaged components, whereas severe or chronic stress shifts the balance toward senescence through excessive organelle loss and metabolic insufficiency [182].

The role of autophagy in cancer has similarly evolved from a uniform tumor-suppressive paradigm to recognition of its context-dependent duality. Early studies demonstrated that autophagy preserves genomic integrity and metabolic homeostasis by eliminating damaged organelles and proteins, whereas autophagy deficiency induces oxidative stress and DNA damage that facilitate oncogenic initiation [172].

However, subsequent studies revealed that hypoxia, nutrient deprivation, and therapeutic stress can repurpose autophagy as a survival mechanism. In established tumors, autophagy supplies ATP, amino acids, and nucleotides and interfaces with p53, RAS, and PI3K/AKT signaling to promote proliferation, invasion, and metastasis [183]. This temporal dichotomy positions autophagy as tumor suppressive during initiation but tumor promoting during progression. For example, stage-specific effects are illustrated in hepatocellular carcinoma, where autophagy eliminates premalignant hepatocytes and maintains hepatic homeostasis by removing defective organelles [184]. Conversely, elevated autophagic activity at later stages correlates with increased tumor burden, metastatic dissemination, and resistance to sorafenib. Similar stage-dependent effects have been reported in preclinical breast cancer models [185].

The oncogenic or suppressive effects of autophagy are further shaped by driver mutations and signals from the TME. Autophagy-dependent metabolites released by stromal cells provide essential substrates for tumor survival and growth. Within the TME, cancer-associated fibroblasts (the dominant stromal population) sustain a proinflammatory phenotype through autophagy-derived ATP and biosynthetic intermediates [186]. Autophagy also remodels the TIME by attenuating CD8^+^ T-cell cytotoxicity, thereby facilitating immune evasion [187].

## Emerging therapeutic strategies targeting aging and cancer

Aging and cancer are distinct yet deeply interconnected biological processes, and their simultaneous pharmacological targeting carries substantial scientific and clinical significance. In this section, we systematically review therapeutic agents directed against aging- and cancer-related pathways, aiming to provide a comprehensive reference for research and translational application. Building on the shared regulatory mechanisms outlined above, we place particular emphasis on strategies capable of concurrently modulating both processes and identify candidate agents with “one drug, two targets” potential. These agents have demonstrated dual efficacy in delaying aging-associated phenotypes and suppressing tumor growth in preclinical studies. The following sections detail these emerging therapeutic strategies.

### Senotherapies

Although aging involves numerous complex mechanisms, senotherapy has emerged as a central therapeutic strategy. Senotherapy aims to eliminate senescent cells or modulate their phenotype, thereby delaying age-related physiological decline and the onset of age-associated diseases. Current approaches can be broadly classified into three categories: senolytic agents, senomorphic modulators, and multimodal regimens (Table 1).

**Table 1:** Senotherapies (Senolytics and Senomorphics): Targets, Research Stage and Key Findings

Strategies	Type	Agent	Target	Model/Trial	Outcome
Senolytics	BCL-2 inhibitor	Navitoclax	BCL-2/BCL-xL	MS mouse model	Improving outcomes and survival [188]
		Venetoclax	BCL-2	Zebrafish model	Ameliorating chronic inflammation [189]
		Foselutoclax	BCL-xL	Phase I trial, NCT04537884	Good tolerability in advanced DME [190]
	p53-targeted drug	FOXO4-DRI	Interaction between p53 and FOXO4	Pulmonary hypertension mouse model	Exhibiting pulmonary hemodynamic alterations [191]
		UBX0101	Interaction between p53 and MDM2	Osteoarthritis mice model	Reducing oxidative protein stress [192]
		RG-7112	Interaction between p53 and MDM2	Back pain mouse model	Reducing low back pain and SASP factor release [193]
	Natural product	Fisetin	Sirtuin 6	Phase I/II trial, NCT04210986	Not reported
		Ellagic acid	SIRT1	Male Wistar rat	Improving renal function in rats [193]
	BRAF inhibition	SB590885	Mitochondrial metabolism	Human diploid fibroblast	Increasing cellular proliferation but decreasing senescent phenotypes [194]
	Galactose derivatives	SSK1	Senescent cell	Aged AD mouse model	Improving cognitive function in aged AD mice [195]
		Nav-Gal	Senescent cell	Senescent hTNBC cell	Eliminating senescent hTNBC cells [196]
	CAR T cells	uPAR-directed CAR T cells	T cell	Aged mouse model	Improving exercise capacity in physiological aging [197]
		NKG2D-CAR T cells	T cell	Aged mouse model	Eliminating naturally occurring senescent cells [198]
Senomorphics	mTOR pathway inhibitor	Rapamycin	mTOR	Phase I trial, NCT01649960	A decreasing trend in β-galactosidase activity [199]
	NF-κB inhibitor	Metformin	NF-κB	nAMD mouse model	Reducing retinal vascular leakage [200]
		SR12343	IKK/NF-κB	Aged mouse model	Reducing senescence markers [201]
		BAY 11–7082	NF-κB	Mice model	Inhibiting senescence in HT22 hippocampal and GT1-7 GnRH neuronal cells [202]
	p38 MAPK inhibitor	SB203580	p38 MAPK	Leydig cell model	Increasing Tyr182 phosphorylation of P38 [203]
		BIRB 796	p38 MAPK	T cell	Reducing proinflammatory cytokines [204]
	ATM inhibitor	KU55933	ATM	Corneal endothelial cell	Restoring DNA repair in G2/M phase and alleviating senescence [205]
		KU60019	ATM	Sepsis mouse model	Alleviating myocardial injury [206]
	Natural products	Kaempferol	ER stress	Drosophila	Inhibiting the hyperproliferation of aging intestinal stem cells [207]
		ITE	mTOR, MAPK, and IκBα phosphorylation	Replicative senescent human dermal fibroblast	Suppressing the inflammatory response [208]
		Curcumin	NF-κB	NCT05761327	Reducing disease activity and inflammation [209]
		Resveratrol	NF-κB	NCT05761327	Reducing disease activity and inflammation [209]
	Cardiac glycosides	Ouabain	IL-1β and TNF-α	Aβ1–42-induced microglial cell	Improving PI3K/AKT signaling pathway activation [210]
	SASP inhibitors	Simvastatin	IL-1, IL-6, and IL-8Anakinra	Vascular smooth muscle cell	Reducing SASP and ROS production [211]
		Anakinra	IL-1	Phase II trial, NCT01300650	Decreasing high-sensitivity C-reactive protein [212]
Combination approaches	Senolytic + Senolytic	D + Q	Senescent cell	Phase II trial, NCT04313634	Increasing the percentage of bone formation marker P1NP at week 2 [213]
		Dasatinib + ellagic acid	Senescent cell and SIRT1	Lung fibroblasts MRC-5 cell	Increasing caspase activity [166]
	Senomorphic + Senomorphic	KU-60019 + Y-27632	ATM and ROCK	Human diploid fibroblast	Promoting G2/M transition via FOXM1 activation [214]
	Senolytic + Senomorphic	Dasatinib + resveratrol	Senescent cell and NF-κB	Senescent human lung fibroblasts MRC-5 cell	Increasing caspase activity [166]
		SB590885 and SB203580	BRAF and p38 MAPK	Human dermal fibroblast	Reducing ROS [215]

Table abbreviations. AD, Alzheimer’s disease; DME, diabetic macular edema; ER, endoplasmic reticulum; hTNBC, human triple-negative breast cancer; MS, multiple sclerosis; nAMD, neovascular age-related macular degeneration; ROS, reactive oxygen species. Data sources: ClinicalTrials. gov (https://clinicaltrials.gov/)

Senolytics selectively eliminate senescent cells, with established examples including dasatinib plus quercetin (D + Q), B-cell lymphoma 2 (BCL-2) family inhibitors, p53-binding antagonists, and various natural compounds [216]. BCL-2 was the first intrinsic regulator of apoptosis to be identified, and BCL-2 inhibitors have demonstrated robust efficacy in eliminating senescent cells in vivo, a finding widely validated in mouse models [217]. However, the first-generation agent ABT-263 (navitoclax) is associated with substantial adverse effects, including neutropenia and thrombocytopenia. Conversely, the newer-generation compound 753b selectively targets senescent cell antiapoptotic pathways while sparing normal hematopoiesis, thereby avoiding severe hematological toxicity and enabling specific clearance of senescent cells [218]. Notably, treatment with 753b markedly attenuates hepatocellular carcinoma driven by metabolic dysfunction–associated steatotic liver disease, underscoring the close interplay between aging and cancer [219]. Next-generation senolytics are advancing rapidly, and their translational status and supporting evidence are summarized in Table 1. Aging is a dynamic and highly heterogeneous process, and some senescent cell populations express low levels of BCL-2, rendering BCL-2 inhibitors less effective in these contexts [220]. Conversely, senescence-associated β-galactosidase (SA-β-gal) is a widely used marker of senescent cells both in vitro and in vivo. Exploiting this feature, the senolytic prodrug senescence-specific killing compound 1, which is selectively activated by SA-β-gal, effectively eliminates senescent cells in models of post-traumatic osteoarthritis [221]. Similarly, the galactose-modified duocarmycin prodrug induces selective apoptosis of senescent cells in a lysosomal β-galactosidase-dependent manner [222]. Additionally, procyanidin C1 (PCC1) binds directly to EGFR and inhibits multiple downstream signaling pathways, thereby promoting senescent cell clearance [223, 224]. Its antiaging effects have been validated in rodent models and shown to confer geroprotective benefits within the hematopoietic and immune systems. Furthermore, chimeric antigen receptor (CAR) T-cell therapy enables precise targeting and elimination of senescent cells and represents an emerging component of senolytic strategies. For example, Amor et al. identified urokinase plasminogen activator receptor (uPAR) as a senescence-enriched cell-surface protein, and uPAR-specific CAR-T cells efficiently ablated senescent cells both in vitro and in vivo, extending survival in mouse models of lung adenocarcinoma [225]. Likewise, Yang et al. developed CAR-T cells targeting NK group 2 member D (NKG2D) ligands, which effectively eliminate senescent cells in aged mice and elderly non-human primates and improve physical function in aged murine models [198].

Senomorphics suppress pathological SASP without eliminating senescent cells. These agents act through pathways such as mTOR, NF-κB, p38 MAPK, and ataxia telangiectasia mutated (ATM) and include various natural and synthetic small molecules (Table 1) [226]. Taking NF-κB as an example, BAY 11–7082 inhibits IL-6 release in senescent HMC3 cells by blocking NF-κB signaling [227]. Metformin exerts broad effects on aging and has been proposed as a potential senomorphic agent. In a mouse model of neovascular age-related macular degeneration, metformin suppressed retinal neovascularization but simultaneously promoted retinal fibrosis [200]. Moreover, a double-blind, randomized, placebo-controlled trial (MET-PREVENT) demonstrated that metformin did not improve 4-m walking speed in older adults at risk of sarcopenia (p = 0.96) [228]. These findings indicate that a comprehensive and context-specific evaluation of metformin’s utility in aging interventions is required. Recent studies have also shown that PDK4 is strongly upregulated with aging and that its inhibition reduces DNA damage and attenuates SASP production [103]. Direct neutralization of SASP components represents an additional senomorphic strategy, with proof-of-concept evidence demonstrated in cellular models [229]. Simvastatin has been shown to reduce expression of proinflammatory cytokines, including IL-1α, IL-1β, IL-6, IL-8, and CXCL-1, in vivo, functioning as a broad-spectrum SASP inhibitor [211]. It also ameliorates mitochondrial dysfunction and suppresses senescent cell burden. Therapeutic agents targeting key SASP mediators therefore represent promising candidates for delaying aging. These include IL-1–targeting agents such as anakinra, canakinumab, and rilonacept; anti–tumor necrosis factor-α (TNF-α) agents such as infliximab, adalimumab, and etanercept; and IL-6–targeting agents such as tocilizumab and siltuximab [230–232]. Recent studies have confirmed that infliximab, etanercept, and tocilizumab exhibit significant senostatic activity. Anakinra has completed Phase II clinical trials, in which reductions in high-sensitivity C-reactive protein (hsCRP) levels were observed in both younger and older patients with heart failure; however, no significant difference in hsCRP reduction was detected between age groups, underscoring the need for larger, adequately powered studies [212].

Combination strategies integrate senolytics and/or senomorphics to enhance therapeutic efficacy. In addition to the conventional D + Q regimen, the senolytic combination of dasatinib and ellagic acid has demonstrated synergistic clearance of senescent cells in cellular models [166]. Combination approaches using two senomorphic agents have also shown promising feasibility in preclinical studies. Yang et al. reported that co-inhibition of ATM and Rho-associated protein kinase promoted proliferation of senescent human diploid fibroblasts, producing a stronger pro-proliferative effect than either agent alone [214]. Additionally, combination strategies that pair senolytic and senomorphic agents have exhibited pronounced senescence-inhibitory effects. For example, dasatinib combined with resveratrol enhanced apoptosis in senescent human lung fibroblasts [166]. Similarly, co-administration of the BRAF inhibitor SB590885 and the p38 MAPK inhibitor SB203580 reduced ROS levels, restored mitochondrial function, and alleviated senescence-associated phenotypes [215].

Notably, nanoparticle-based drug delivery systems have demonstrated substantial efficacy and represent a highly promising strategy for enhancing future therapeutic outcomes. For example, encapsulation of PCC1 within a yolk–shell nanoparticle delivery system markedly increases its intestinal concentration and extends the maximum lifespan of *D. melanogaster* by 30.8% [233]. Similarly, delivery of uPAR-targeted CAR mRNA via PL40 lipid nanoparticles has been shown to effectively ameliorate uPAR-associated liver fibrosis and rheumatoid arthritis [234].

### Therapeutic targets in cancer

Advances in cancer research have established various systematic, target-driven therapeutic strategies. Major current directions include modulation of epigenetic regulation, targeting TMMs, activation of antitumor immune responses, regulation of autophagic balance, and metabolic intervention. The corresponding targeted therapeutic strategies are discussed below.

Epigenetic anticancer strategies primarily target four enzymatic families: DNA methyltransferases (DNMTs), HMTs, histone demethylases, and histone deacetylases (HDACs). The nucleoside analogs azacitidine and decitabine, which are already approved for the treatment of hematologic malignancies, reactivate silenced tumor-suppressor genes through DNMT inhibition [235]. Next-generation oral agents, such as zebularine and procaine, are currently in preclinical and early-phase clinical development and are designed to overcome limitations related to stability and drug delivery [236]. At the level of histone methylation, selective enhancer of zeste homolog 2 (EZH2) inhibitors, including GSK-J1 and GSK-J4, suppress H3K27me3 deposition and have demonstrated strong anticancer activity in both solid tumor and hematologic malignancy models [237]. The small-molecule antagonist OICR-9429 disrupts WD repeat domain 5 (WDR5)–mixed lineage leukemia (MLL) interaction and has shown therapeutic potential in CCAAT enhancer binding protein alpha (*CEBPA*)-mutant AML [238]. Notably, GSK-J1 also inhibits the H3K27 demethylases lysine demethylase 6 A (KDM6A) and KDM6B [239]. Additionally, HDAC inhibitors such as vorinostat and belinostat have received regulatory approval for the treatment of T-cell lymphomas and other hematologic cancers [240].

Telomerase-targeted agents are advancing through a multi-tiered clinical development pipeline. The first-in-class oligonucleotide inhibitor GRN163L (imetelstat) met its primary endpoint in the phase III IMerge trial by significantly reducing transfusion dependence in patients with relapsed or refractory lower-risk MDS and has received Food and Drug Administration (FDA) approval as the first telomerase-directed therapy [241]. BIBR1532, a non-nucleosidic small-molecule inhibitor, sensitizes NSCLC cells to irradiation by inducing ferroptosis and activating the cyclic GMP-AMP synthase (cGAS)–stimulator of interferon genes (STING) pathway [242]. The telomerase-recognized nucleoside analog 6-thio-2'-deoxyguanosine disrupts telomere integrity and induces immunogenic cell death, producing potent anticancer effects in hepatocellular carcinoma and melanoma models [243]. G-quadruplex (G4) stabilizers, including TMPyP4, telomestatin, and BRACO19, lock telomeric G4 structures into nonextendable conformations, thereby inhibiting telomere elongation [244]. Furthermore, an eight-arm polyethylene glycol (PEG)-ylated "nanooctopus" construct physically entangles G4 DNA to induce telomere crisis [245]. From an immunotherapeutic perspective, hTERT-derived peptide vaccines such as GV1001, GX301, and Vx-001 bind MHC molecules to prime adaptive antitumor immune responses [246].

To address the challenges posed by TIS, a combinatorial anticancer strategy known as the “one–two punch” approach has been developed [247]. This strategy initially employs radiotherapy, chemotherapy, or immunotherapy to induce tumor cell death or senescence, followed by selective clearance of senescent cells using targeted agents. Dou et al. demonstrated that combined treatment with a PDK4 inhibitor and the chemotherapeutic agent mitoxantrone reduced senescence marker expression and tumor burden [103]. Similarly, Wu et al. reported that combining the valosin-containing protein (VCP) inhibitor CB-53399 with the senolytic agents ABT-263 and conatumumab effectively eliminated senescent pancreatic ductal adenocarcinoma cells and enhanced overall anticancer efficacy [248].

Immunotherapy, which reactivates host antitumor immunity, has become a core pillar of oncology alongside surgery, radiotherapy, and chemotherapy. Immune checkpoint blockade, exemplified by inhibitors of PD-1/PD-L1 and CTLA-4, has demonstrated durable efficacy across multiple solid malignancies [249, 250]. Furthermore, next-generation immune targets (including LAG-3, TIM-3, TIGIT, and members of the B7-H family) are advancing rapidly through preclinical and early-phase clinical development [251–253]. In the field of adoptive cell therapy, CD19-targeted CAR-T cells have achieved transformative clinical responses in acute lymphoblastic leukemia (ALL) and non-Hodgkin lymphoma, while CAR-NK cells have shown favorable safety profiles and promising efficacy in early studies of hematologic malignancies [254]. CAR-engineered mesenchymal stem cells have also demonstrated effective tumor infiltration and cytotoxic activity in preclinical models of glioblastoma and Ewing sarcoma [255]. Cancer vaccine strategies include cellular, viral, peptide, and nucleic acid–based formulations, with dendritic cell vaccines and prophylactic human papillomavirus vaccines already approved and clinically effective [256, 257]. Additionally, oncolytic virotherapy using herpes simplex virus, adenoviral, and measles virus vectors has led to formulation of licensed products such as talimogene laherparepvec (T-VEC) and Oncorine [258]. Accumulating preclinical and clinical evidence further indicates that combining oncolytic viruses with immune checkpoint inhibitors significantly enhances antitumor efficacy [259].

The ambivalent role of autophagy in cancer renders its therapeutic targeting both promising and challenging. On one hand, autophagy activation eliminates damaged organelles and proteins, thereby preserving cellular integrity and suppressing tumor initiation. On the other hand, excessive or sustained autophagy can confer metabolic advantages that enhance tumor cell stress resistance and promote disease progression. Consequently, current research efforts are exploring both autophagy agonists and antagonists, strategically positioned at distinct nodes of the autophagic pathway (Fig. 4, Table 2). Among autophagy-activating agents, temsirolimus has received FDA approval for the treatment of advanced renal cell carcinoma, and everolimus is approved for late-stage renal, breast, and other solid tumors [278], validating autophagy activation as a viable therapeutic strategy. Building on these clinical successes, next-generation agents, including AMPK agonists, Beclin-1 activators, and LC3 enhancers, are entering antineoplastic development pipelines. In parallel, autophagy inhibition has also demonstrated anticancer efficacy. ULK-100 induces apoptotic cell death by blocking autophagy and the pentose phosphate pathway [265], while chloroquine and hydroxychloroquine inhibit cytoprotective autophagy and markedly enhance radiosensitivity [279]. Beyond targeting ULK1 and lysosomal function, additional components such as VPS34, PI3K, and LC3 are emerging as promising therapeutic targets. However, their tumor-specific roles and reliable predictive biomarkers remain to be fully elucidated.

**Table 2:** Autophagy-targeted agents for cancer therapy

Phase	Type	Autophagy	Agent	Model/Trial	Outcome
Initiation	mTOR inhibitor	Activation	Rapamycin	Human pancreatic cancer cell	Inhibition of mTOR activation enhances antitumor activity [260]
			Temsirolimus	MDA-MB-231 cell	Enhancing CD8 + T-cell–mediated antitumor immunity [261]
			Everolimus (EVE)	Phase II trial, NCT03032406	Targeting dormant RTCs to deplete minimal residual disease [262]
	AMPK agonist	Activation	Aspirin	Mouse model	Enhancing chemotherapeutic efficacy through aspirin administration [263]
			Resveratrol	Oral cancer cell	Inducing autophagy via resveratrol to inhibit oral cancer cell proliferation [264]
	ULK1 inhibitor	Inhibition	ULK-101	*KRAS* mutant lung cancer cell	Inhibition of ULK1 to sensitize cells to nutrient stress [265]
			SBI-0206965	Endothelial cell	Improving vascular function [266]
			MRT68921	HepG2 and Huh-7 cell	Improving antiproliferative and proapoptotic effects of cinobufagin [267]
Phagophore nucleation	VSP34 inhibitor	Inhibition	VPS34-IN1	AML cell	Inducing apoptosis [268]
			SAR405	NSCLC tumor cell	Enhancing cisplatin-induced cell death [269]
	Beclin-1 activator	Activation	Piceatannol	Gastric cancer cell	Inhibition ofcell proliferation and colony formation [270]
			Quercetin	Lung cancer cell	Inhibition of cell survival in a dose-dependent manner [271]
	PI3K inhibitor	Inhibition	Wortmannin	Breast cancer cell	Inducing apoptosis [272]
Phagophore elongation and maturation	LC3 inhibitor	Inhibition	DC-LC3in-D5	HeLa cell	Impaired formation of autophagic structures and reduced degradation of autophagic substrates [273]
			LC3in-C42	Cell	Inhibiting autophagy [274]
	LC3 enhancer	Activation	Quercetin	Lung cancer cell	Inhibition ofcell survival in a dose-dependent manner [271]
			Ursolic acid	BALB/c mouse model	Inhibition ofcancer proliferation [275]
Fusion	Lysosome inhibitor	Inhibition	Monensin	Glioblastoma cell	Inducing apoptosis [276]
			Bafilomycin A1	Human colon cancer sample	Decreasing Na +/K + ATPase activity and increasing basal Mg2 + ATPase activity [277]

Table abbreviations. RTC, residual tumor cell. Data sources: ClinicalTrials. gov (https://clinicaltrials.gov/)

Exploiting the highly specific metabolic rewiring of malignant cells, an expanding array of interventions targeting key metabolic nodes has demonstrated substantial antineoplastic potential. Given the systemic reprogramming of glucose, glutamine, and fatty acid flux within the TME, inhibitors of glycolysis, glutaminolysis, and de novo lipogenesis have emerged as central strategies in precision oncology. Muhammad et al. comprehensively mapped these metabolic vulnerabilities and synthesized recent preclinical advances and therapeutic rationales [111]. At the level of nutrient sensing and signal transduction, the growth hormone (GH)/IGF-1, mTOR, and AMPK pathways coordinately regulate neoplastic nutrient acquisition and utilization. Small-molecule modulators of these pathways are under active development and preclinical evaluation, with early-phase clinical trials already indicating reductions in tumor burden and delayed disease progression, as reviewed by Zhang et al. [8]. Beyond metabolic interventions, direct targeting of oncogenic drivers such as *KRAS* and *MYC* is also advancing. Notably, bromodomain and extra-terminal domain (BET) family inhibitors disrupt bromodomain-containing protein 4 (BRD4) binding at the *MYC* promoter, thereby attenuating *MYC* transcription and producing potent anticancer effects across multiple solid and hematologic malignancy models [280].

Furthermore, exosomes and nanoparticles function as precision delivery systems that transport therapeutic payloads selectively to tumor cells, thereby enhancing efficacy while limiting systemic toxicity [281, 282]. Although natural exosomes can serve as drug carriers, they predominantly accumulate in the liver and spleen, possess complex and heterogeneous compositions, and consequently exhibit limited therapeutic efficiency. Conversely, engineered exosomes incorporate targeting moieties that enable preferential tumor accumulation, substantially enhancing the antitumor efficacy of radiotherapy, chemotherapy, and immunotherapy while also mitigating drug resistance [281]. Nevertheless, clinical translation of engineered exosomes remains challenging due to obstacles related to manufacturing standardization and precise payload control. Nanoparticle-based platforms likewise facilitate tumor-selective drug delivery, enhance radiosensitization, and improve drug bioavailability. Notably, nanobodies, owing to their small size, high stability, and low immunogenicity, are advancing the field of nano-immunotherapy and possess the ability to cross the blood–brain barrier, offering a promising therapeutic strategy for central nervous system malignancies and other brain disorders [283, 284].

### Therapeutic opportunities at the aging–cancer nexus

Aging and malignancy converge on multiple molecular hubs that are amenable to therapeutic intervention, providing a strong biological rationale for the “one drug, two targets” concept. Table 3 systematically summarizes representative agents that act on these shared pathways. Collectively, these agents demonstrate the translational potential of simultaneously modulating aging- and cancer-related mechanisms and offer practical guidance for future clinical research.

**Table 3:** Convergent therapeutics targeting shared pathways in aging and cancer

Pathway	Type	Drug	Outcome (Model/Trial Phase)	
			Senescence	Cancer
Senescence	BCL-2 inhibitor	Venetoclax	Ameliorating chronic inflammation (zebrafish model) [189]	Using marketed drugs for the treatment of hematologic tumors
		Navitoclax	Reducing the expression of age-related plasma proteins such as IL-23R (mice) [285]	Reporting SVR_35_, TSS_50_, and BMF improvement rates of 26.5%, 30%, and 33%, respectively, at week 24 (Phase II trial, NCT03222609) [286]
	Natural product	Fisetin	Not reported (Phase I/II trial, NCT04210986)	Prevents neurons from taking in extracellular vesicles containing m6A-modified RNAs and suppressing the excessive innervation and progression of PDAC tumours (mice) [287]
		Ellagic acid	Improving renal function (rats) [193]	Ellagic acid-protein nano-complex inhibits tumor growth by reducing the intratumor bacteria and inhibiting histamine production (mice) [288]
		Kaempferol	Inhibiting the hyperproliferation of aging intestinal stem cells (cells) [207]	Exhibiting anti-proliferative and anti-migratory effects in time- and dose-dependent manners (Cal27 cells) [289]
		Curcumin	Reducing disease activity and inflammation (NCT05761327) [209]	Reducing cell viability(prostate cancer (cells) [290]
		Resveratrol	Reducing disease activity and inflammation (NCT05761327) [209]	Facilitating the cancer cell ferroptosis (mice) [291]
	mTOR pathway	Rapamycin	A decreasing trend in β-galactosidase activity (Phase I trial, NCT01649960) [199]	Increasing naïve T cell count in low-grade prostate cancer patients with Rapamycin capsules and demonstrating a favorable safety profile (Phase I) [292]
	ATM inhibitor	KU55933	Restoring DNA repair in G2/M phase and alleviating senescence (cells) [205]	Iinhibiting tumor growth and metastasis in mammary tumors through inhibition of GLUT1 translocation and vimentin expression (mice) [293]
		KU60019	Alleviating myocardial injury (sepsis mice) [206]	Suppressing hsa-miR-1273 g-3p level and elevating DGAT1 level (SKOV3 cells) [294]
	Cardiac glycosides	Ouabain	Improving PI3K/AKT signaling pathway activation (Aβ1–42-induced microglial cells) [295]	Compromising cell viability and inducing cell apoptosis (TNBC cells) [296]
	SASP inhibitors	Simvastatin	Reducing SASP and ROS production(vascular smooth muscle cells) [211]	Inducing ferroptosis by inhibiting ILF3 in GC cells and enhanced the killing effect of activated CD8 + T cells on GC cells (GC cells) [297]
Epigenetics	DNMT inhibitor	Decitabine	Improving vascular repair after sepsis (mice) [298]	Enhancing immunogenicity in KRAS-LKB1 mutant lung cancer (mice) [299]
		Azacitidine	Improving hypertrophic cardiomyopathy (cells) [300]	Enhancing anticancer immune responses in CRC immunotherapy (Phase II) [301]
	NAD^+^	NMN	Improving blood–brain barrier leakage associated with aging (cells) [302]	Inhibiting cancer initiation (mice) [303]
		NR	Alleviating brain injury following cerebral hemorrhage (mice) [304]	Eliminating precancerous cells (cells) [305]
	HDAC inhibitor	Vorinostat	Improving cognitive dysfunction in AD (mice) [306]	Enhancing prognosis in pancreatic cancer (cells) [307]
		Belinostat	Improving cell senescence and photoaging caused by ultraviolet radiation (mice) [308]	Treating relapsed or refractory peripheral T-cell lymphoma (approved) [309]
Immunity and inflammation	Anti- PD-1	RMP1-14	Improving aging-related kidney and liver phenotypes (mice) [310]	Improving survival in diffuse large B-cell lymphoma (mice) [311]
	CAR T	NKG2D- CAR T	Eliminating senescent cells in aged mice and non-human primates (mice and non-human primates) [198]	Exhibiting high anticancer efficacy against NKG2DL-positive cervical cancer cell lines (cells) [312]
Microbiota	FMT	——	Reversing aging phenotypes in the gut, eyes, and brain (mice) [313]	Improving survival in patients with microsatellite-stable metastatic CRC (Phase II) [314]
	Probiotics	Lactobacillus rhamnosus	Extending lifespan in Caenorhabditis elegans (Caenorhabditis elegans) [315]	Enhancing anticancer immunity (mice) [316] Delaying cancer onset (mice) [317]
		Bifidobacterium	Slowing immune senescence (cells) [318]	Promoting anticancer immunity and enhancing the efficacy of anti-PD-L1 (mice) [318]
	Prebiotics	Inulin	Increasing gut microbiota diversity (mice) [319]	Enhancing chemotherapy sensitivity (mice) [320]
Metabolism	HK inhibitor	2-DG	Mimicking caloric restriction in aging models (rats) [321]	Alleviating cancer-induced muscle atrophy (mice) [322]
	FAO inhibitor	Trimetazidine	Improving muscle strength in aged mice (mice) [323]	Reducing the viability of pancreatic cancer cells (cells) [324]
	GT inhibitor	Phloretin	Exerting antioxidant effects (mice) [325]	Inducing apoptosis in human glioblastoma cells (cells) [326]
		PGG	Preventing photoaging (mice) [327]	Inducing cytotoxicity and reducing proliferation in colon cancer cells (cells) [328]
	FAS inhibitor	C75	Preventing senescence induction and reducing SASP secretion (cells) [131]	Exhibiting cytotoxicity against prostate cancer cell lines (cells) [329]
Autophagy	AMPK activator	Metformin	Alleviating aging-related inflammation (T cells) [330]	Inhibiting cancer cell proliferation (mice) [331]
	mTOR inhibitor	Rapamycin	Improving skeletal muscle function (mice) [332]	Treating metastatic pancreatic cancer (cells) [333]
		Temsirolimus	Reversing HGPS cell phenotypes (cells) [334]	Enhancing chemotherapy sensitivity (cells) [335]
	VPS34 inhibitor	SAR405	Partial inhibition improves exercise capacity and extends healthspan (mice) [336]	Reducing proliferation in renal cancer cells (cells) [337]

Table abbreviations. AD, Alzheimer’s disease; BMF, bone marrow fibrosis; CAF, cancer-associated fibroblast; CRC, colorectal cancer; DNMT, DNA methyltransferase; FAO, fatty acid oxidation; FAS, fatty acid synthesis; FMT, fecal microbiota transplantation; GC, gastric cancer; GT, glucose transport; HGPS, Hutchinson-Gilford progeria syndrome; HK, hexokinase; HSC, hematopoietic stem cell; NAD^+^, nicotinamide adenine dinucleotide; NR, nicotinamide riboside; SVR_35_, ≥ 35% spleen volume reduction; TSS_50_, ≥ 50% reduction in total symptom score. Data sources: ClinicalTrials. gov (https://clinicaltrials.gov/)

Cellular senescence is a shared hallmark of aging and cancer and, in principle, renders senotherapies intrinsically suitable for dual targeting. Although antitumor research has not yet encompassed all senotherapeutic agents, many have demonstrated direct antitumor activity or the ability to enhance therapeutic efficacy [338]. For example, venetoclax, a BCL-2 inhibitor, effectively eliminates senescent cells and is an established treatment for multiple hematological malignancies. Similarly, navitoclax targets both BCL-2 and BCL-xL to achieve senescent cell clearance and has emerged as a promising agent for blood cancers [285]. In patients with MF who exhibited suboptimal responses to ruxolitinib monotherapy, the addition of navitoclax resulted in 26.5% of patients achieving a ≥ 35% reduction in spleen volume from baseline (SVR_35_) at week 24, 30% achieving a ≥ 50% reduction in total symptom score (TSS_50_) at week 24, and 33% of evaluable patients demonstrating a 1–2 grade improvement in bone marrow fibrosis [286]. Although anemia and thrombocytopenia were observed, these adverse events were manageable. Based on these Phase II clinical findings, the results of the ongoing Phase III TRANSFORM-1 trial evaluating navitoclax in MF are highly anticipated. Additionally, several inhibitors targeting ATM signaling and components of the SASP have shown dual potential in clearing senescent cells while suppressing tumor growth (Table 3).

At the epigenetic level, the DNMT inhibitor azacitidine is already used clinically for hematologic malignancies, where it reactivates silenced tumor-suppressor genes through demethylation. In aged mice, azacitidine also restores FoxM1-driven regenerative programs in the vascular endothelium, markedly accelerating tissue repair following sepsis [235, 298]. Similarly, HDAC inhibitors such as vorinostat and belinostat confer cross-disease benefits. The EZH2 inhibitor tazemetostat further exemplifies the dual utility of targeting HMTs, suppressing aberrant H3K27me3 deposition in follicular lymphoma while attenuating senescence-associated functional decline in preclinical aging models [339].

Dysregulation of the nicotinamide adenine dinucleotide (NAD)^+^ metabolic axis represents another convergent vulnerability between aging and cancer. In aged tissues, declining NAD^+^ levels compromise DNA repair capacity and epigenetic regulation, whereas cancer cells exploit elevated nicotinamide phosphoribosyltransferase expression to sustain NAD^+^ availability, thereby supporting poly(ADP-ribose) polymerase (PARP)-mediated DNA repair and high glycolytic flux [340, 341]. Supplementation with the NAD^+^ precursor nicotinamide mononucleotide (NMN) replenishes NAD^+^ levels in aged mice, reactivates sirtuin 1 (SIRT1)/SIRT3 signaling, improves diastolic cardiac function, and extends lifespan [342]. In immunocompetent cancer models, NMN administration prevents macrophage senescence, preserves T-cell effector function, and suppresses tumor initiation [303]. Owing to its low toxicity toward non-malignant cells, NAD^+^ augmentation represents a pragmatic therapeutic strategy for simultaneously targeting aging-associated decline and early-stage oncogenesis.

Rebalancing the immune–inflammatory axis represents a paradigmatic example of convergent, pathway-based intervention. PD-1/PD-L1 signaling is upregulated in aging tissues, where it drives T-cell exhaustion and chronic inflammation, and is simultaneously exploited by cancers to evade immune surveillance [343, 344]. Accordingly, PD-1 blockade enables concurrent targeting of senescence and malignancy. The anti–PD-1 antibody RMP1-14 reverses podocyte senescence and extends glomerular lifespan in aged murine kidneys [310] while also significantly improving survival in models of Myd88- and BCL2-driven diffuse large B-cell lymphoma [311]. Additionally, in an oral squamous cell carcinoma model, RMP1-14 alleviates cancer-related pain by inhibiting the spinal TNF-α pathway [345]. Beyond checkpoint inhibition, CAR-T-cell platforms further expand the scope of immunotherapeutic strategies. As summarized in Table 3, NKG2D-CAR-T cells effectively eliminate senescent cells in aged mice and non-human primates, improve metabolic profiles, and exhibit potent antitumor activity in both AML and ALL models [198, 346].

Autophagy and microbiota modulation further exemplify dual-action therapeutic synergies. Low-dose rapamycin extends median lifespan in aged mice while concurrently suppressing tumor growth in pancreatic cancer xenograft models [333]. Fecal microbiota transplantation reframes the gut microbiome as a programmable therapeutic platform: transplantation of microbiota from young donors reverses senescence markers in aged mice and enhances anti-PD-1 efficacy in patients with refractory melanoma [313, 347]. Probiotic and prebiotic interventions similarly demonstrate dual potential by attenuating aging-associated phenotypes while augmenting anticancer immune responses, as summarized in Table 3.

Targeting metabolic enzymes with small molecules now offers a clear translational pathway. For instance, the fatty acid oxidation inhibitor trimetazidine enhances skeletal muscle strength in aged mice while simultaneously reducing pancreatic cancer cell viability [323]. Phloretin, a glucose transporter inhibitor, attenuates collagen deposition in models of pulmonary fibrosis and induces apoptosis in glioblastoma cells [326]. Additional metabolic targets and candidate compounds are summarized in Table 3. Although most of these agents remain at the preclinical stage, their dual targeting of aging- and cancer-related pathways provides a valuable lead library for the rational design of future combination therapies.

Nanomedicines, particularly versatile all-in-one platforms, hold significant promise for aging and cancer therapy because of their capacity to integrate multiple treatment modalities. Jibira et al. developed curcumin–piperine nanoparticles that demonstrated superior efficacy in reducing prostate cancer cell viability compared with single-agent formulations [290]. Another example is HA@IR780@KU55933@BSA (HIKB), a nanodrug designed for triple-negative breast cancer [348]. By co-delivering the photosensitizer IR780 and the ATM kinase inhibitor KU55933, HIKB achieves tumor-specific accumulation and synergistically induces ROS production, mitochondrial dysfunction, and DNA damage in vivo.

Beyond pharmacological interventions, health-oriented lifestyles centered on dietary modulation and regular physical activity are increasingly recognized as convergent deterrents of both aging and cancer. Caloric restriction reduces systemic and intratumoral insulin/IGF-1 signaling, activates AMPK, and suppresses mTOR, thereby extending lifespan and lowering cancer incidence in rodent and primate models [349, 350]. Extensive animal studies and clinica–epidemiological evidence further indicate that physical exercise attenuates inflammatory signaling in endothelial and stromal compartments and mitigates oxidative stress, delaying aging, reducing the risk of multiple cancer types, and improving clinical outcomes [351, 352]. Importantly, the aging-modulating and anticancer effects of diet and exercise extend beyond metabolic regulation, arising from multidimensional biological networks that encompass dynamic epigenetic remodeling, enhanced autophagy, restored redox homeostasis, and optimization of the immune microenvironment [8].

## Challenges and future directions

The relationship between cellular senescence and cancer represents one of the most paradoxical phenomena in tumor biology. Clinically, aging is an independent risk factor for increased cancer incidence. Although senescence is characterized by proliferative arrest and metabolic decline, whereas cancer exhibits unchecked growth and increased metabolic demand, the underlying molecular pathways governing these processes are deeply interconnected and frequently overlapping.

However, the relationship between aging and cancer is not linear; a greater degree of aging does not necessarily correspond to an increased cancer risk. Several discordant phenomena contribute to this unresolved gray zone. For instance, mutational landscapes exhibit nonlinear associations with oncogenesis, with NOTCH mutations displaying variable incidence and prognostic implications across different tumor types [353]. ncRNAs and RNA modifications are likewise highly context dependent. As an example, insulin-like growth factor 2 mRNA-binding protein 2 (IGF2BP2) is downregulated in aged HSCs but overexpressed in AML, where its expression is associated with poor prognosis [354, 355]. Telomere biology further adds complexity: although short telomeres often increase cancer risk and are observed in approximately 70% of tumors, exceptionally long telomeres also confer elevated risk for several major cancers, exemplifying the telomere–cancer paradox [356].

Overall, the relationship between aging and cancer is characterized by substantial biological complexity (Fig. 1). This review systematically elucidates the molecular correspondence between aging and cancer through the framework of their core hallmarks, with particular emphasis on nine pivotal hallmark pairs whose interactions can be broadly classified as synergistic promotion, antagonistic suppression, or context-dependent duality. Importantly, these classifications reflect a consensus based on the current body of evidence rather than fixed or universally applicable rules. As experimental approaches continue to evolve and mechanistic insights deepen, these relationships are likely to be further refined and redefined.

Among the nine hallmark pairs examined, genomic instability–associated genetic variants have been investigated most extensively, both historically and systematically. The prevailing consensus holds that gene mutations exacerbate genomic instability, thereby compromising genomic integrity and establishing a molecular substrate for tumor initiation. However, recent findings from Stefánsson et al. have revealed a cancer-preventive role for specific rare germline variants [357]. Notably, combined loss-of-function and missense variants in *PPP1R15A* reduce breast cancer risk by 53%, while an increased burden of such variants in *AURKB* is associated with a 16% reduction in overall cancer risk. These observations prompted the authors to propose a broader hypothesis: each aging hallmark may exert dual effects on cancer development, with the balance between tumor-suppressive and tumor-promoting functions varying across hallmarks and biological contexts. The relative weighting of these opposing effects determines whether a given hallmark pair is classified as synergistic, antagonistic, or dualistic. Within this framework, many apparent contradictions in aging–cancer relationships can be coherently reconciled.

The current delineation of synergistic, antagonistic, and dualistic relationships between the core hallmarks of aging and cancer remains inherently provisional and subject to refinement as research progresses; nevertheless, this mechanistic framework, grounded in available evidence, already provides a robust biological rationale for the “one drug, two targets” therapeutic paradigm [358]. Accordingly, Sect. "Therapeutic opportunities at the aging–cancer nexus" systematically summarizes candidate agents that act on shared pathways, offering concrete directions for implementing this strategy. Despite its promise, clinical translation of the “one drug, two targets” paradigm faces several key challenges. Much of the existing evidence is derived from animal models, and agents that have advanced to clinical testing largely remain in early-phase trials, highlighting the need to accelerate translational and clinical validation. Furthermore, interventions such as NAD^+^ modulation and autophagy regulation may exert pro-tumorigenic effects if applied at inappropriate stages, doses, or intervals [359], underscoring the importance of precise therapeutic calibration. Moreover, although the potential of exosome-based and nanotechnology-driven delivery systems is increasingly recognized, continued optimization of delivery routes is required. Compared with conventional administration methods, rationally engineered nanocarriers can substantially enhance in vivo drug accumulation and improve therapeutic efficacy [233].

In summary, aging and cancer are not governed by a simple zero-sum antagonism but instead exhibit a complex interplay of synergy, antagonism, and context-dependent duality across genomic, epigenetic, immune, metabolic, and microenvironmental dimensions. The nine hallmark pairs not only provide a biological explanation for the increased incidence of cancer in older adults but also establish a conceptual framework for the development of unified therapeutic strategies.

## Data Availability

Not applicable.
